# A novel workflow correlating RNA-seq data to *Phythophthora infestans* resistance levels in wild *Solanum* species and potato clones

**DOI:** 10.3389/fpls.2015.00718

**Published:** 2015-09-17

**Authors:** Itziar Frades, Kibrom B. Abreha, Estelle Proux-Wéra, Åsa Lankinen, Erik Andreasson, Erik Alexandersson

**Affiliations:** Department of Plant Protection Biology, Swedish University of Agricultural SciencesAlnarp, Sweden

**Keywords:** *Solanum*, *Phytophthora infestans*, RNA-seq, *R*-genes, potato, resistance

## Abstract

Comparative transcriptomics between species can provide valuable understanding of plant-pathogen interactions. Here, we focus on wild *Solanum* species and potato clones with varying degree of resistance against *Phytophthora infestans*, which causes the devastating late blight disease in potato. The transcriptomes of three wild *Solanum* species native to Southern Sweden, *Solanum dulcamara, Solanum nigrum*, and *Solanum physalifolium* were compared to three potato clones, Desiree (cv.), SW93-1015 and Sarpo Mira. Desiree and *S. physalifolium* are susceptible to *P. infestans* whereas the other four have different degrees of resistance. By building transcript families based on *de novo* assembled RNA-seq across species and clones and correlating these to resistance phenotypes, we created a novel workflow to identify families with expanded or depleted number of transcripts in relation to the *P. infestans* resistance level. Analysis was facilitated by inferring functional annotations based on the family structure and semantic clustering. More transcript families were expanded in the resistant clones and species and the enriched functions of these were associated to expected gene ontology (GO) terms for resistance mechanisms such as hypersensitive response, host programmed cell death and endopeptidase activity. However, a number of unexpected functions and transcripts were also identified, for example transmembrane transport and protein acylation expanded in the susceptible group and a cluster of Zinc knuckle family proteins expanded in the resistant group. Over 400 expressed putative resistance (*R*-)genes were identified and resistant clones Sarpo Mira and SW93-1015 had ca 25% more expressed putative R-genes than susceptible cultivar Desiree. However, no differences in numbers of susceptibility (*S-*)gene homologs were seen between species and clones. In addition, we identified *P. infestans* transcripts including effectors in the early stages of *P. infestans*-*Solanum* interactions.

## Introduction

Wild *Solanum* species are alternative hosts for pathogens of cultivated potato, which is a crop attacked by many different microorganisms. Local populations of these often grow in close proximity of potato fields. Compared to cultivated potatoes, which are not well adapted to the local environment and also might have lost important genetic variation through domestication, wild species could be a source for desirable traits to breed disease-resistant varieties of the crop.

The oomycete *Phytophthora infestans* is the causing agent of late blight leading to annual losses in potato worth billions of US dollars (Haverkort et al., 2008). In the last few decades, many resistance (*R-*)genes against this devastating pathogen have been successfully transferred from various wild *Solanum* species to potato (Vleeshouwers et al., 2011; Rodewald and Trognitz, 2013). However, the pathogen contains fast-evolving effector genes localized in a highly dynamic region of its genome (Haas et al., 2009), enabling it to quickly overcome the *R*-gene based resistance in potato.

Functional stacking of *R*-genes has been employed to achieve resistance (Zhu et al., 2012) and new cultivars such as Sarpo Mira have a wider mix of *R*-genes introgressed (Rietman et al., 2012). The emergence of next-generation sequencing techniques enable inexpensive characterization of expressed *R-*genes also in non-model plants (Draffehn et al., 2013; Yang et al., 2014). Apart from *R*-gene conferred resistance, studying *Solanum* lineage-specific adaptive responses and comparing wild *Solanum* and potato interactions with pathogens can provide valuable insights into the underlying molecular events (Draffehn et al., 2013; Du et al., 2013; Ali et al., 2014).

Three wild *Solanum* species, which are potential hosts for *P. infestans*, grow in Sweden. Bittersweet nightshade (*S. dulcamara*) is a diploid semi-woody, outcrossing, perennial climber, while the di- tetra- or hexaploid European black nightshade (*S. nigrum*) and diploid hairy nightshade (*S. physalifolium*) are self-compatible, annual weeds. Populations of these species grow in the proximity of potato cultivations but differ in sensitivity to *P. infestans*. *S. dulcamara* and *S. nigrum* are only rarely infected by *P. infestans* but still can harbor the pathogen (Deahl et al., 2004, 2010), while *S. physalifolium* has been reported to be a sensitive host, which might influence pathogen aggressiveness in potato (Andersson et al., 2003; Grönberg et al., 2012). *S. dulcamara* has previously been shown to vary in resistance toward *P. infestans*, and *R*-genes, *Rpi-dlc1* and *Rpi-dlc2*, have been mapped (Golas et al., 2010, 2013). A comprehensive characterization of the *S. dulcamara* transcriptome and genome-wide genetic map have also been presented (D'Agostino et al., 2013).

Coevolution between plants and their natural enemies is believed to be an important driver of biological diversity (Burdon and Thrall, 2009). Variation in transcriptomes among wild species can therefore be expected to be largely influenced by natural selection on defense to pests and pathogens, in addition to selection in relation to beneficial biotic interactions and abiotic factors (Oh et al., 2014). In crop species, on the other hand, artificial selection caused by the domestication process and subsequent breeding have been shown to have direct effect on the transcriptome (Swanson-Wagner et al., 2012; Bellucci et al., 2014).

The aim of this study was to characterize defense responses against *P. infestans* in wild *Solanum* species and to identify factors successfully conferring resistance. Using RNA-seq we therefore generated leaf transcriptome atlases of individuals of *S. dulcamara* (accession Sd 3:6), *S. nigrum* (accession Sn 4:3) and *S. physalifolium* (accession Sp 2:4) and compared these to the transcriptomes of three tetraploid potato clones: Sarpo Mira and SW93-1015, which exhibit strong *P. infestans* resistance (Ali et al., 2012), and Desiree (cv.), which is susceptible. We have previously explored the response to infection with *P. infestans* in these potato clones by gene expression microarrays and proteomics (Ali et al., 2014).

**Figure 1:**
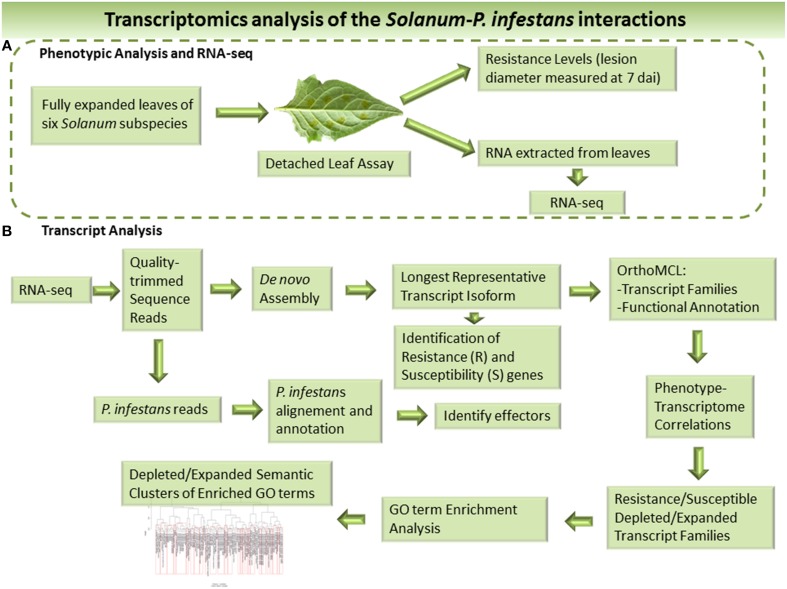
**Overview the workflow used to identify resistance and susceptible factors in the host and effectors of the infecting pathogen based on RNA-seq**. **(A)** Phenotypic analysis and RNA Sequencing (RNA-Seq). **(B)** Bioinformatic analysis of the RNA-seq data and transcriptomes.

In order to capture lineage-specific events reflecting adaptive evolution and conferring resistance, we used a novel workflow incorporating a correlation-based method to identify clusters either expanded or depleted in transcript numbers dependent on the level of *P. infestans* resistance of the wild *Solanum* and potato clones studied. An overview of the workflow used is presented in Figure 1. We used class vectors describing the level of plant resistance either qualitatively, i.e., resistant vs. susceptible, or quantitatively, i.e., based on an experimentally determined resistance gradient. Clusters were analyzed functionally by gene ontology (GO) and clusters populated with *R-*genes studied in more detail. We also identified 100's of putative *R*-genes in the three wild *Solanum* species and three potato clones. Furthermore, *P. infestans* transcripts representing RXLR effectors, Crinklers (CRN) and elicitins were detected.

## Materials and methods

### Plant material, growth conditions, and resistance assay

Seeds of wild *Solanum* species (*S. dulcamara*, accession Sd 3:6; *S. nigrum*, accession Sn 4:3; and *S. physalifolium*, accession Sp 2:4) were collected from natural habitats in Southern Sweden. Wild *Solanum* and potato clones Desiree (cv.), Sarpo Mira, and SW93-1015 were grown in a growth chamber set to 20°C, 16:8 h light:dark cycle and 70% relative humidity. Based on a detached leaf resistance assay with *P. infestans* (strain SE-03058) performed as described in Ali et al. (2012) one individual from each wild *Solanum* species and the three potato clones were selected for transcriptome profiling. Detached leaves without inoculation, hereafter referred to as at 0 h after inoculation (hai), at 24 and 48 hai were frozen in liquid nitrogen for RNA extraction.

For the resistance assay, detached leaf assays with five leaves per each clone/species plant were inoculated with *P. infestans*. Long and short axes of the lesion on each leaf were measured 7 days after inoculation, and then averaged to show the infection level quantitatively in each clone/species. Based on the lesion size, a gradient (from most resistant to most susceptible) was determined. The resistance gradient was statistically validated by a Kruskal-Wallis test with the PCMCR R-package (Pohlert, 2014) for pairwise multiple comparison between mean ranks of the lesion size.

### RNA isolation and sequencing

RNA was isolated from fully expanded leaves detached from 6-week-old plants and incubated in conditions used for the resistance assay. For the potato clones leaves were collected before infection and at 24 hai, as described in Ali et al. (2014), whereas the wild *Solanum* leaves were harvested at 0, 24, and 48 hai.

The RNA extractions were performed using the RNeasy Plant Mini kit (Qiagen GmbH, Hilden, Germany). Samples were DNase treated and cleaned using the Qiagen RNA cleanup kit. RNA concentration and purity (260/280 nm > 1.8) was determined with an ND-1000 NanoDrop (Wilmington, USA) and integrity of the samples were analyzed with an Experion™ (Bio-Rad Laboratories, Hercules, USA). Equimolar amount of RNA from the leaves at 0, 24, and 48 hai was pooled for each wild *Solanum* before sequencing using Illumina HiSeq 2000 at Beijing Genomics Institute (BGI, Beijing, China). The RNA-seq data has been deposited as *de novo* assembled FASTA files in ArrayExpress: E-MTAB-2953.

### Transcript assembly, identification of orthologous groups

The raw reads were processed with Trimmomatic v 0.3 (Bolger et al., 2014) in order to remove low-quality bases. The *P. infestans* reads were removed from the *Solanum* datasets by mapping the reads on the *P. infestans* genome using TopHat2 (version 0.8b) (Kim et al., 2013). Three independent *de novo* assemblies were then built from the remaining paired-end reads with Trinity (r2013_08_14) (Grabherr et al., 2011) using default parameters. To estimate the assemblies' quality bowtie2 (v. 2.1.0) was used to calculate the overall alignment rate of the quality-trimmed reads of the transcriptome assemblies. For each Trinity component a representative sequence was chosen, based on length, and Transdecoder was then used to obtain the longest protein sequence. The three protein sequence FASTA files obtained were then used as an input for OrthoMCL (Li et al., 2003) along with proteomes from *Arabidopsis thaliana* (TAIR10), *S. lycopersicum* (ITAG2.3), *O. sativa* (RGAP7), *S. tuberosum* group Phureja (PGSC3.4) and the International Tomato Annotation Group (ITAG1.0). Three additional proteomes of *S. tuberosum* clones Desirée (cv.), Sarpo Mira and SW93-1015 derived from either uninfected or *P. infestans* infected (isolate SE-03058) transcriptomes assembled as described in Ali et al. (2014) were finally added. OrthoMCL (v2.0.3) (Li et al., 2003) was run on these 11 proteomes with default parameters.

### Identification of expanded/depleted OrthoMCL clusters

In order to identify expanded or depleted OrthoMCL clusters, prototype vectors holding the differences in transcript numbers for the resistant and susceptible species and clones qualitatively (i.e., resistant vs. susceptible species and clones) or quantitatively (i.e., related to a gradient vector based on the results of the resistance assay, described above) were constructed. For both analyses a matrix of transcript numbers for each OrthoMCL clusters of all 6 clones/species was generated (Table S1). Additionally, two prototype vectors were created reflecting the qualitative and quantitative susceptibility-resistance relationship between the plants. Thereafter association between paired samples was tested by Spearman's rho statistics (test for rank-based measure of association) between each OrthoMCL cluster and each prototype vector in order to determine positive or negative associations. This gave four *p*-values for each OrthoMCL cluster that measure quantitative and qualitative positive and negative associations (Table S1), representing expanded and depleted clusters (*p* < 0.05), respectively.

### Functional annotation of the expanded and depleted OrthoMCL clusters

GO enrichment analysis of the expanded and depleted OrthoMCL clusters was done by a hypergeometric test using GOEAST (Zheng and Wang, 2008) with default parameters. In order to create an annotation file, all OrthoMCL clusters were assigned GO terms based on the genomic sequences belonging to the cluster. In this way the potato clones and *Solanum* wild species sequences inherited the annotations of the genome sequences dependent on the OrthoMCL structure. The following functional annotations were used: TAIR10 (Arabidopsis), RGAP7 (rice), ITAG2.4. For the two potato gene models PGSC and ITAG GO terms were used as assigned in Amar et al. (2014). Related processes were displayed as significant bootstrap clusters (Suzuki and Shimodaira, 2006) of GO term semantic similarities calculated using the GOSimSem package (Yu et al., 2010).

### Identification of plant resistance (R)-, susceptibility (S)-genes, and *P. infestans* effectors

Two complementing ways were used to identify *R*-genes. Firstly, a discriminatory psp-gen MEME (Bailey et al., 2009) analysis identified 20 NB-LRR protein sequence motifs (Table S3) using the same positive NB-LRR and negative non-NB-LRR sequence training sets as in Jupe et al. (2012) keeping their classification of the motifs. MAST (Bailey et al., 2009) was then used to search for occurrences of the 20 motifs in the wild *Solanum* species and potato clone transcript sequences. Transcripts were considered candidate NB-LRRs if the reported MAST *E*-values were lower than the smallest *E*-value observed for a member of the negative training set in Jupe et al. (2012) (i.e., *E* < 8.5e-24). The NB-LRRs were classified as CNLs or TNLs based on the presence of previously classified MEME motifs (Table S4). Alternatively, BLASTP searches (parameters: -*evalue 1e-5 -outfmt 6 -max_target_seqs 1*) with 112 reference resistance genes deposited in the Plant Resistance Genes database (Sanseverino et al., 2012) were performed (Table S5). For the BLASTP results a sequence cut-off of 30% identity and 30% coverage was applied and then classified according to the domain classification of their homologs in the Plant Resistance Genes database. The resulting *R*-genes were validated with a Pfam domain search (Finn et al., 2014) (Table S5). In order to identify specific *R*-gene members BLASTP and BLASTN searches (parameters: *-evalue 1e-5 -outfmt 6 -max_target_seqs 1*) were performed with R1, R2, R3, and Rpi-blb1 genes as baits (Table S6). *S*-genes were identified by BLASTP and BLASTN (parameters: *-evalue 1e-5 -outfmt 6 -max_target_seqs 1*) with *A. thaliana* mitogen-activated protein kinase 4 (MAPK 4), *A. thaliana* homoserine kinase 1 and 6 (dmr1, dmr6), and the Barley mlo together with its Capsicum and *S. tuberosum* homologs as baits (Table S7).

To identify effectors of *P. infestans* acting during the infection process in different species, sequence reads of each wild *Solanum* and potato clone were mapped and aligned using (TopHat v2.0.8b) on the *P. infestans* strain T30-4 reference genome (http://www.broadinstitute.org/). Unique reads mapped to the *P. infestans* reference genome were counted using the htseq-count program (HTSeq-0.5.4p3). The *P. infestans* annotation file from The Broad institute (downloaded 08/07/2014; http://www.broadinstitute.org/) was used to identify the potential RxLR effectors, Crinklers (CRNs) and elicitins among the *P. infestans* reads (Table S8).

### Handling and visualization of data

R (v3.0.3) and Perl (v5.18.2) were used to handle, parse, transform and statistically analyze the data. Venn diagrams was produced by VENNERABLE (Swinton, 2011). Qlucore Omics Explorer (v3.0) was used for construction of principle component analysis (PCA).

## Results and discussion

### *De novo* transcriptome assembly

Between 54 and 90 Mb clean paired-end sequence reads of *S. dulcamara, S. nigrum, S. physalifolium*, Desiree, Sarpo Mira, and SW93-1015 were *de novo* assembled using Trinity (Table 1). The reads were of high and consistent quality for all species and clones (Figure S1). The amount of clean reads should be sufficient to generate in-depth, representative assemblies of leaf transcriptome atlases for the species and clones studied here, since 20 million reads for tissue samples has been shown to be sufficient for non-model mammal species (Francis et al., 2013). There was no big variation in the GC content of the assemblies, 40–41% (Table 1), which is similar to the content in the coding domain sequences predicted from the potato genome (43%).

**Table 1:** **Summary of transcriptome assemblies for three wild ***Solanum*** species and three potato clones**.

		***S. nigrum***	***S. dulcamara***	***S. physalifolium***	***Sarpo Mira***	***SW93-1015***	***Desiree***
Total paired end reads (^*^10^6^) GC (%)		27.6	26.7	25.8	52.1	52.4	51.9
		40.4	40.3	41.0	40.2	40.0	40.3
Contigs using all transcripts	Total assembled bases (^*^10^6^)	54.2	64.6	62.9	71.4	75.7	89.8
	Average length	520	695	644	663	571	740
	N50	797	1245	1095	1258	1011	1402
	Median length	299	366	352	331	294	369
	Number trinity genes	80,316	70,912	66,632	75,995	81,471	80,825
	Number trinity transcripts	104,172	92,992	82,219	107,758	132,454	121,377
Contigs using longest isoform	Total length (10^6^)	39.4	40.3	37.7	40.3	39.1	45.4
	Average length	491	568	565	531	480	562
	N50	736	961	931	956	813	1012
	Median length	281	308	313	272	251	291
	Number of transcripts	27,988	26,156	27,968	25,045	23,925	24,267
Overall alignment rate (%)		91.7	95.2	94.2	92.8	94.5	92.7

The N50 value for the contigs, generated using the longest representative, were larger than the values recently reported for closely related species *S. melongena* and *S. torvum* (Yang et al., 2014). This could be due the longer read length (90 bp) of the RNA-seq data we used and is indicating a high quality of the assemblies with a high level of represented leaf transcripts. In all the assemblies, the contig N50 value generated using the longest isoform was smaller (736–1012 bp) than the N50 derived from all the assembled transcripts (797–1402 bp) (Table 1). The lower N50 value in the assembly using only the longest isoform could be associated with the reduced total sequence length (Table 1) due to the removal of redundant transcripts. However, commonly used reference-free measures including median contig length, number of contigs and N50 can be misleading as measures of assembly quality. Therefore, we mapped all the transcripts back to the assemblies and found that overall alignment rates were constant and high for all six assemblies between 91.7 and 95.2% (Table 1).

The number of transcripts identified is within the range of what can be expected in plant leaf transcriptomes, granted that both tomato and potato have ca 35,000 predicted genes (Tomato Genome Consortium, 2012). There was a slightly higher number of transcripts based on the longest representative in the wild *Solanum* species (26,156–27,988) than in the potato clones (23,925–25,045), which is in line with previous reports on decreased diversity of gene expression in domesticated crops (Bellucci et al., 2014) (Table 1).

### Resistance levels of species and clones

Both the *S. nigrum* (Sn 4:3) and *S. dulcamara* (Sd 3:6) accessions used in this study are resistant, whereas the *S. physalifolium* (Sp 2:4) accession is susceptible to the *P. infestans* strain. Potato clones responded to *P. infestans* infection as expected. Desirée (cv.) is a well-studied food potato with a high degree of susceptibility to *P. infestans* infection. Sarpo Mira is a potato cultivar that recognizes five different effectors from *P. infestans* and shows a classical HR reaction in response to inoculation with the pathogen (Rietman et al., 2012). SW93-1015 is a breeding potato clone, which is resistant to Swedish *P. infestans* populations with reduced HR expansion (Ali et al., 2012).

**Figure 2:**
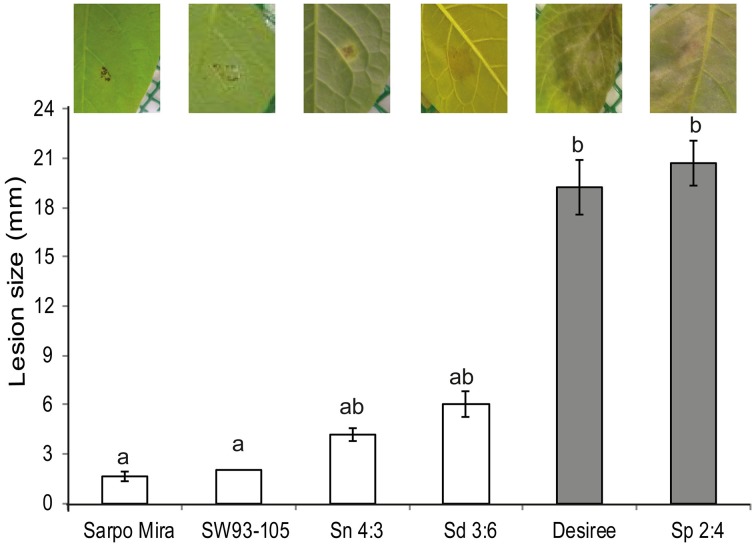
**Detached leaves from plants of three wild ***Solanum*** species, (***S. nigrum***, Sn 4:3; ***S. dulcamara***, Sd 3:6; and ***S. physalifolium***, Sp 2:4), and potato clones (Sarpo Mira, SW93-1015, and Desiree) inoculated with ***Phytophthora infestans*****. The pathogen grew on the susceptible Desiree and Sp 2:4 (gray bars) whilst its growth was restricted to the inoculation site on the resistant ones (white bars). Pictures were taken at 7 days after inoculation (dpi). Error bars indicate standard error of the mean. Bars that are not connected by the same letter are significantly different (*P* < 0.05).

We were only able to detect visible macroscopic symptom of the *P. infestans* inoculation after 48 hai as noted for potato in a review by Fry (2008). The 45 hai is referred as biotrophic phase (Vleeshouwers et al., 2000) whilst the 72 hai is considered the start of the necrotrophic phase of the pathogen (Birch et al., 2003). Consequently, RNA was isolated either up to 24 hpi (potato clones) or 48 hpi (wild *Solanum* species) before macroscopic lesions occurred in any of the species or clones and before the onset of the necrotrophic phase. Therefore, the samples in this study cover only the biotrophic phase of the *P. infestans* infection. Although our analysis groups the samples as either as susceptible or resistant to make general findings, the samples should not be regarded as proper biological replications. That sort of comparison is difficult, if not impossible, to accomplish, since progression of infection inevitably varies between species and clones with varying resistance levels. Based on the resistance assay using detached leaves a resistant to susceptible gradient was established in the following order: Sarpo Mira, SW93-1015, *S. nigrum, S. dulcamara*, Desiree, and *S. physalifolium* (Figure 2). Applying the Kruskal-Wallis test on mean rank of the lesion size [$\chi_{\left(5\right)}^{2}=26.1$, *P* < 0.01] showed a resistance difference and the grouping was in line with the gradient.

In plant resistance biology detached leaf assays are widely used even if they do not reflect the physiology of the intact plant or environmental conditions in the field. In more in depth studies it is necessary to relate the results between the two systems to each other. Here, we have only studied the infection progress and transcriptomes under detached leaf conditions, to which the species and clones might react differently. This also overlooks reactions in other environments such as field conditions. However, the correlation-based method identifying expanded and depleted transcript families based on resistance level is dependent on relating the phenotypic response during the same conditions, justifying the capturing the transcriptomes during detached leaf conditions. Future more comprehensive studies should take the genotype x environment interactions into account by testing different environments and pathogen strains and their influence on resistance levels and transcriptome compositions. The overall workflow of the present study is available in Figure 1.

### Comparative analysis of OrthoMCL cluster composition

**Figure 3:**
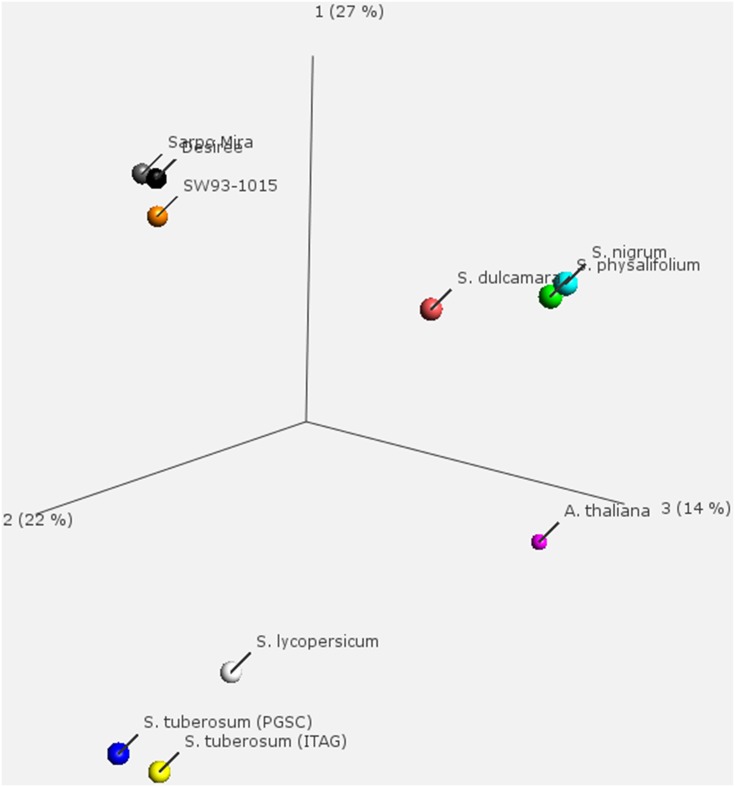
**OrthoMCL principle component analysis (PCA)**. The PCA is based on number of transcripts for *Solanum nigrum, S. dulcamara*, and *S. physalifolium*, Desiree, Sarpo Mira, SW93-1015 as well as number of genes for *S. tuberosum* (PGSC or ITAG gene models), *S. lycopersicum*, and *A. thaliana*.

**Figure 4:**
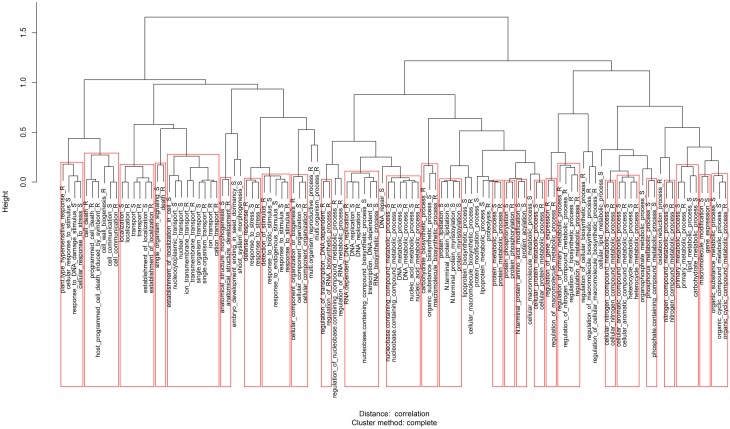
**Clades of GO enriched terms for Biological Process (Siddappa et al., 2014) of the expanded and depleted OrthoMCL clusters determined quantitatively according to the resistance gradient (GO enriched terms based on the qualitative analysis is available in Figure S2)**. R and S denotes GO terms enriched in the resistant or susceptible groups, respectively. Broad, unspecific GO terms have been removed.

Using the predicted proteomes of the 11 species and clones in OrthoMCL, 38,890 clusters were generated, in which leaf transcripts of Desiree, Sarpo Mira, SW93-1015, *S. nigrum, S. dulcamara*, and *S. physalifolium* were represented in 27,979 (Table S2). In the analyses of the transcriptomes only the longest representative transcript was used to avoid allelic variants caused by different polyploidy, mis-assemblies and sequencing errors (D'Agostino et al., 2013). The OrthoMCL cluster structure was analyzed by PCA (Figure 3) and GO term enrichment analysis (Figure 4; Figure S2). Displaying the OrthoMCL clusters constructed by transcripts of Desiree, Sarpo Mira, SW93-1015, *S. nigrum, S. dulcamara*, and *S. physalifolium* and genes *S. tuberosum* (PGSC or ITAG gene models), *S. lycopersicum* and *A. thaliana*, there are clear differences between transcript and gene data (principle component 1, PC1) as can be expected since not all genes are present in the leaf transcriptomes studied here. A division of *S. tuberosum* clones and wild *Solanum* follows PC2. Furthermore, OrthoMCL clusters expanded or depleted as for numbers of transcripts were identified and either linked to a gradient depending on the degree of resistance to *P. infestans* (quantitative analysis) or grouped as resistant vs. susceptible (qualitative analysis). These analyses found 143 and 1291 OrthoMCL clusters significantly (*p* < 0.05) expanded quantitatively and qualitatively, respectively, and 249 and 525 significantly depleted quantitatively and qualitatively, respectively (Table S1). In the qualitative analysis as we contrast two susceptible with four resistant species and clones and therefore we have unequal sequence amounts for the resistant and susceptible entities, 158.8 and 77.7 Mb, respectively, which could influence results. However, this unequal relation is not seen in the quantitative analysis and we find a substantial overlap of 86% in the clusters identified by both analyses showing coherence between both approaches.

### Accession- and species-specific OrthoMCL clusters

**Figure 5:**
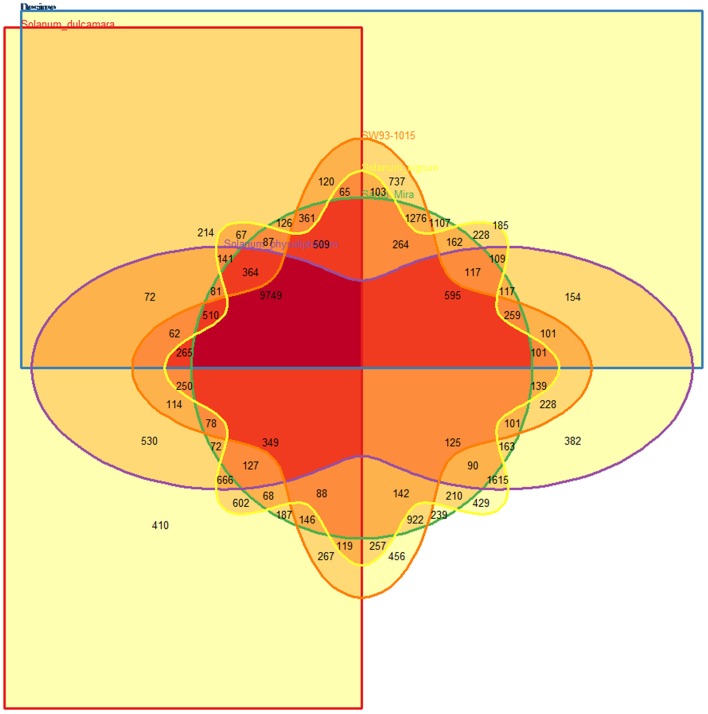
**OrthoMCL cluster overlap between ***Solanum nigrum*** (yellow), ***S. dulcamara*** (red), and ***S. physalifolium*** (lilac), Desiree (blue), Sarpo Mira (green), SW93-1015 (orange) based on detected transcripts**.

Under detached leaf conditions *S. dulcamara* had slightly fewer transcripts represented in the OrthoMCL clusters compared to the others, which had representatives in roughly 2/3 of all OrthoMCL clusters (Table 2). With 456 OrthoMCL clusters only containing SW93-1015 transcripts, this clone had the highest number of unique clusters (Table 2; Figure 5). However, since these observed differences in expressed transcripts are from detached leaf assays solely, to which the species/clones might react differently to the incubation condition, results on species and clone-specific features should be seen as preliminary until the transcriptomes from several environments and most importantly from field and natural habitat conditions have been obtained.

**Table 2:** **OrthoMCL cluster summary**.

	***S. nigrum***	***S. dulcamara***	***S. physalifolium***	**Sarpo Mira**	**SW93-1015**	**Desiree**
Represented (%)	65	60	64	67	67	66
Unique	429	410	382	239	456	185

*Distribution of the transcripts based on percentage of OrthoMCL clusters with transcripts members and number of unique OrthoMCL cluster for respective species and clone*.

A GO enrichment analysis of the unique OrthoMCL clusters (Table S9) found apoptotic process, response to oxidative stress, chitin binding and chromatin binding corresponding to clusters of transcripts with sequence similarity to *Rpi-vnt1*, other NBS-LRRs, 42 kDa chitin-binding protein and MYB factors in SW93-1015. The *Rpi-vnt1* gene from *S. venturii* confers late-blight resistance in both potato and tomato (Foster et al., 2009). Interestingly, in Sarpo Mira the GO term terpenoid biosynthetic process was uniquely enriched. Among the wild *Solanum* enrichment of defense response to fungus and regulation of peptidase activity in *S. dulcamara*, peptidase activity acting on L-amino acid peptides and histone modification in *S. nigrum* and cell wall modification and epidermis development in *S. physalifolium* are noteworthy with regards to plant resistance. Enriched GO terms of unique OrthoMCL clusters specific for each accession are found in Table S9.

### Transcript clusters expanded in the resistant group

The qualitative (resistant vs. susceptible species and clones) and quantitative (resistance gradient) analyses identified OrthoMCL clusters significantly expanded in the resistant group. GO term enrichment analysis of expanded groups identified terms such as protein phosphorylation, defense response, plant-type hypersensitive response, host programmed cell death induced by symbiont and aspartic-type endopeptidase activity (Figure 4; Figure S2). These are all associated to plant defense and were therefore studied in more detail.

A number of expanded transcript clusters were populated by transcripts encoded by resistance genes showing the usefulness of the method. Examples include several clusters with the NBS-LRR resistance family members and a large cluster of R1 Late blight resistance homologs. This specific OrthoMCL cluster (WILD0000054) had 13 and 11 expressed members in SW93-1015 and Sarpo Mira, respectively, and 10 members in *S. nigrum*, whereas susceptible Desiree and *S. physalifolium* only had seven and six members, respectively. Many of the clusters with NBS-LRR representatives were specific to the *Solanum* species lacking transcript members from Arabidopsis and rice. The protein of the NBS-LRR resistance gene (PGSC0003DMP400054560) shows high sequence identity (60%) to a potato Bacterial spot disease resistance protein 4 (*Bs4*) homolog. In tomato, BS4 (Solyc05g007850) gives resistance against *Xanthomonas campestris* causing bacterial leaf spots (Schornack et al., 2004). In *S. demissum* the BS4 homolog is flanking the functional R1 locus (Kuang et al., 2005).

An expanded defense-related cluster contained Glutamate-gated kainate-type ion channel receptor subunits (*GluR5s*), which are ligand-gated non-selective cation channels. Overexpression in Arabidopsis delays fungal infection of fungal pathogen *Botrytis cinerea* (Kang et al., 2006) and have been shown to mediate MAMP-induced calcium influx in *A. thaliana* (Mark et al., 2011).

Some expanded transcript clusters in the resistant group without clear connections to defense were identified. For example, a small cluster of Zinc knuckle family proteins, which has been shown to have RNA binding and splicing activities (Lopato et al., 1999), and *Diminutivo 1* involved in Brassinosteroids biosynthesis and lignin formation (Hossain et al., 2012).

### Transcript clusters expanded in the susceptible group

GO term enrichment analysis associated clusters expanded in the susceptible group to terms such as lipoprotein biosynthetic process, signal transduction, protein acylation, transmembrane transport and nitrogen biosynthesis (Figure 4; Figure S2). An example of a putative susceptibility factor present in one of the clusters is the cytokinin-regulated kinase 1 (*CRK1*). In tobacco (Schäfer and Schmülling, 2002), CRK1 of tobacco is a class I receptor kinase located in transmembrane, hypothesized to be involved in hormone signaling.

Transcripts related to ATPase activity, such as the vacuolar H+-pumping ATPase required for salinity stress tolerance in potato (Queirós et al., 2009), was overrepresented in the susceptible group. Histidine kinase is a vital component in signaling transduction and used by plants to sense and respond to biotic and abiotic stresses were also expanded. As were transcripts for sugar and carbohydrate transport, which could be integral part of the network distributing photosynthetically assimilated carbon and transcripts for development of embryo, reproductive structure, fruit and seed.

Even if fewer than in the resistant group, some GO terms associated to plant defense responses were enriched also in the susceptible group, and could be components of the basal disease resistance related to PAMP-triggered immunity (PTI) present also in susceptible hosts (Jones and Dangl, 2006). One cluster was populated with Avr9/Cf-9 rapidly elicited protein 189 (*ACRE189*). The *ACRE189* encodes widely conserved F-box/leucine rich proteins, which are up-regulated upon elicitation by the race-specific avirulence peptide Avr9 in tomato and tobacco, and plays a crucial role in HR development (van den Burg et al., 2008). GO enrichment identified transcripts of serine/threonine protein kinases, and a member of this protein kinase class, Avr9/Cf-9 inducing kinase 1 (*ACIK1*), is important in the Cf9/Avr9- and Cf4/Avr4-mediated HR response against the tomato leaf mold fungus *Cladiosporium fulvum*, which contains the Avr9 (Rowland et al., 2005). Another expanded transcript contained zinc finger binding proteins, which could be integral components of some resistance genes in potato against potato virus X (PVX) and nematodes (Gupta et al., 2012).

### Comparison of expressed *R*-gene homologs

The *R*-gene encoded *R*-proteins, most of which are nucleotide-binding-leucine-rich-repeat (NB-LRR) type, recognize avirulence effectors of the pathogen during the infection process (Hogenhout et al., 2009; Thomma et al., 2011; Giraldo and Valent, 2013) resulting in a hypersensitive response (HR), which restricts expansion of the pathogen. Most of the cloned NB-LRR proteins from *Solanum* species belong to the N-terminal coiled coil-nucleotide-binding site-leucine-rich repeat (CNL), Toll interleukin1 receptor-nucleotide-binding site-leucine-rich repeat (TNL) and receptor-like protein (RLP) classes (van Ooijen et al., 2007). A motif based search identified 438 NB-LRR type genes in the sequenced potato genome of *S. tuberosum* Group Phureja genome (Jupe et al., 2012). Still, Phureja is susceptible to *P. infestans* in spite of the large number of NB-LRR genes, and thus, these genes seem ineffective against *P. infestans*. Previously identified functional *R*-genes, belonging to the largest class CC-NB-LRR, conferring resistance against *P. infestans* includes the *R1* from *S. demissum* (Ballvora et al., 2002), *Rpi-sto2* from *S. stoloniferum* (Champouret, 2010), *Rpi-blb1* and *Rpi-blb2* of *S. bulbocastanum* (van der Vossen et al., 2003, 2005), and the *Rpi-vnt1* of *S. venturii* (Foster et al., 2009; Pel et al., 2009).

*R*-gene homologs sharing NB-LRR domains in the wild *Solanum* were identified by the MEME/MAST analysis as in Jupe et al. (2012), and by blasting 112 reference *R*-genes from the Plant Resistance Genes database (Sanseverino et al., 2012) (Tables S4, S5). The identified motifs include CNLs, TNLs, RLPs, three truncated classes (kinase, which has a kinase domain and an extracellular leucine-rich repeat, NL and TN) and “Other” with no typical resistance-related domains, but which still have been described as conferring resistance through different molecular mechanisms, e.g., *asc-1* (Table 3).

**Table 3:** **Summary of expressed R-gene homologs in ***Solanum*** species**.

	***S. nigrum***	***S. dulcamara***	***S. physalifolium***	**Sarpo Mira**	**SW93-1015**	**Desiree**
CNL^a^-RGdb^b^	9	13	7	12	14	14
CNL-MEME	34	36	28	54	64	43
kinase	1	1	1	1	1	1
NL	1	1	2	2	2	1
RLK^c^	7	8	8	7	8	7
RLP^d^	4	7	7	4	8	6
TN	0	1	1	2	1	1
TNL^e^-RGdb	1	1	2	3	4	1
TNL-MEME	3	4	4	11	10	4
other	11	11	10	11	11	11
Total	62	69	63	94	107	75
RGdb-MEME CNL overlap	9	13	6	11	14	13
RGdb-MEME TNL overlap	0	1	1	2	2	1

a *CNL, N-terminal coiled coil–nucleotide-binding site–leucine-rich repeat*;

b *RGdb, Resistance Genes database*;

c *RLK, receptor-like kinase*;

d *RLP, receptor-like protein*;

e *TNL, Toll interleukin1 receptor–nucleotide-binding site–leucine-rich repeat*.

The MEME/MAST analysis is powerful in identifying putative R-genes lacking high overall sequence identity with known *R*-genes, but which still contain conserved domains. However, with the training set used this analysis is restricted to NB-LRR protein identification (CNLs or TNLs) in contrast to identification by sequence identity of the whole genes. Furthermore, a MAST search with the 20 MEME motifs identified only 69 of the 112 reference *R*-genes in the Plant Resistance Genes database. The discrepancy between the two methods to find putative *R*-genes justifies the use of both approaches. The validation done with Pfam indeed showed that the vast majority contain the NB-ARC domain, indicative of putative *R*-genes (Tables S4, S5). Figure S3 shows the overlap of orthoMCL clusters containing *R*-genes between *Solanum* wild species and potato clones.

According to the MEME/MAST analysis, we found both TNL and CNL representatives in the transcript assemblies represented by 3–11 and 28–64 putative *R*-genes, respectively (Table 3), reflecting the fact that the largest part of NB-LRR genes belonged to the CNL class in the Phureja genome (Jupe et al., 2012). SW93-1015 had the largest number of expressed putative *R-*gene (107). Overall, SW93-1015 and Sarpo Mira had 45–65% more expressed putative *R*-gene than the wild *Solanum* and 25–43% more than the susceptible cultivar Desirée (Table 3).

Closer analysis of putative *R1, R2, R3* and *Rpi-blb1* representatives based on sequence identity revealed a small number of genes (Table 4; Table S6). Interestingly, *S. dulcamara* and Sarpo Mira, which are resistant against *P. infestans*, harbor unique OrthoMCL clusters for these specific classes of *R*-genes (WILD0014801 and WILD0031982, and WILD0036062, respectively).

**Table 4:** **Summary of expressed putative ***R1, R2, R3***, and ***Rpi-blb1*** genes**.

	***S. nigrum***	***S. dulcamara***	***S. physalifolium***	**Sarpo Mira**	**SW93-1015**	**Desiree**
*R1*	1	1	1	1	1	1
*R2*	3	4	4	4	4	3
*R3*	1	2	1	2	1	1
*Rpi-blb1*	1	1	1	1	1	1
Total	6	8	7	8	7	6

In addition to *R*-genes, we investigated four families of susceptibility (*S*)-gene homologs whose impairment results in disease resistance. For example, a loss-of-function mutation of the SlMlo1 in tomato was recently demonstrated to decrease susceptibility to fungal pathogen *Leveillula taurica* (Zheng et al., 2013). Expect for the classic example of mlo, we investigated the putative expansion of *DMR6, DMR1*, and *MPK4* (Petersen et al., 2000; van Damme et al., 2008, 2009). However, all species and lines had the same number and composition of *S*-genes expressed (not shown). So at least for the four *S*-gene families tested, no link existed between (*S*)-gene transcript composition and *P. infestans* susceptibility.

Our results suggest that it is possible to scan the composition of *R*-genes expressed by RNA-seq and characterize a number of putative *R*-genes from wild *Solanum* species. By focusing on the transcriptome non-expressed genes are eliminated, which are likely to be unimportant in the resistance reaction and thus of little use as novel resources of resistance.

### Analysis of transcription of *P. infestans* genes

Even if our methods where not optimized to identify *P. infestans* transcripts we detected transcripts including effectors expressed during the biotrophic phase of the *P. infestans*-plant interaction. A total of 7769, 2612, 1471, 892, and 73 transcripts from *P. infestans* were identified in inoculated samples from *S. physalifolium, S. nigrum*, SW93-1015, Desiree, and *S. dulcamara*, respectively (Figure S4). However, this number of *P. infestans* transcripts does not follow the resistance gradient. We hypothesize that this can either be due to differences in host resistance mechanisms that prevent the pathogen at different stages of the infection process reflecting different layers of resistance responses or due to low overall detection of *P. infestans* reads, which were substantially lower than plant reads forming only 0.35–0.0003% of total reads detected for each species or clone. Still, these results indicate that RNA-seq studies, if optimized, can be a powerful tool to monitor the progress of *P. infestans* during the infection, not least before macroscopic lesions occur in relation to susceptibility or resistance of the host.

The *P. infestans* transcripts represented putative RxLR effectors, Crinklers (CRNs) and elicitins (Table 5; Table S8). No *P. infestans* transcript was detected in the resistant potato clone Sarpo Mira (Table 5). The *P. infestans* genome consists of 563 RXLR and 196 CRN genes out of the total 17,797 predicted protein coding genes (Haas et al., 2009). We found no less than 134 RXLR effectors of the pathogen in the susceptible *S. physalifolium* (Table 5; Table S8), whereas no *P. infestans* effectors were detected in *S. dulcamara* and Sarpo Mira, indicating an efficient early defense response by both hosts upon perceiving the pathogen associated molecular patterns (PAMPs).

**Table 5:** **Number of expressed ***P. infestans*** effector transcripts identified in each species and clone**.

	**No of *P. infestans* transcripts**	**RXLR**	**Crinklers**	**Elicitins**
*S. physalifolium*	7769	134	77	16
*S. nigrum*	2612	41	15	7
SW-1015	1471	18	14	4
Desiree	892	13	6	2
*S. dulcamara*	73	0	0	0
Sarpo mira	0	0	0	0

The secreted RxLR effector peptide avrblb1 (*ipiO1*; PITG_21388) was detected in all but *S. dulcamara* and Sarpo Mira. Identified in a screening of *P. infestans* effectors in resistant potato clones (Vleeshouwers et al., 2008), the *avrblb1* leads to HR in plants containing the *Rpi-blb1 R*-gene from *S. bulbocastanum*. The *avrblb2* (PITG_04090, PITG_04086, PITG_04085) and *avr2* (PITG_22870, PITG_22870, PITG_08943, PITG_13940) family of secreted RxLR effector peptides were also found during the infection of both *S. nigrum* and *S. physalifolium*. Secreted RxLR effector peptide Avr4 (PITG_07387), which has been studied previously (van Poppel et al., 2008) was only identified in *S. physalifolium*.

Among the identified elicitins were the elicitin-like proteins *INF4* (PITG_21410) and *INF6* (PITG_12556), elicitin precursor *INF1* (PITG_12551) and elicitin *INF2A-like protein* (PITG_12561).

## Conclusions and perspective

Compared to cultivated potatoes, which might have lost genetic variation through domestication, wild relatives could provide a source of traits that can be used to breed disease-resistant cultivated varieties. This study provides a first resource of comparative transcriptomics for three wild *Solanum* species. Furthermore, we have contrasted these transcriptomes to those of three potato clones. In our analysis we identified a number of expanded and depleted transcript families by applying a gradient based on the level of resistance of the species and clones to *P. infestans*. Additionally, we present a number of putative *R-*genes based on sequence identity and presence of specific domains, which could be future candidates in resistance breeding. This study also shows that transcripts in the *P. infestans* and *Solanum* pathosystem can be analyzed simultaneously.

It is an important but challenging task to link phenotypes to transcript profiles. In this study several candidate transcript with an effect on the resistance phenotype were identified, such as the members of the R1 transcript family (WILD0000054) expanded in resistant Sarpo Mira and *S. dulcamara*. Furthermore, two clusters (WILD0014801 and WILD0031982) were found to be populated with an R-gene only present in *S. dulcamara*. The importance of these R-genes should be further tested by generating transgenic plants. There are a number of elements that can be seen as novel in this study and the combination of these certainly is. Firstly, our analysis of the *Solanum* material is based on correlations between resistance phenotypes and transcript family compositions. It has been common to contrast expressional changes between phenotypes focusing on differentially expressed genes ever since the microarray technique was introduced, but using the correlations of phenotype-transcript family compositions remain little explored. Secondly, instead of using genomes as input for OrthoMCL to identify gene families, which is common, transcript sequences determined by RNA-seq was used to identify expanded transcript families associated to resistance or susceptibility. Lastly, in order to identify processes associated to resistance or susceptibility of infection with *P. infestans*, the network structure created by OrthoMCL was used to transfer annotations from annotated transcriptomes to the unannotated wild *Solanum* transcriptomes. This enables to do GO term enrichment analyses and subsequent clustering by semantic similarity to improve visualization and help interpretation.

The rapidly decreasing cost of sequencing is increasing the number of available transcriptomes from species and accessions with varying level of resistance to biotic as well as abiotic stresses, and will, thus, enable more comprehensive comparative analyses on the RNA-level. In addition, new emerging sequencing techniques with longer read length will lessen the obstacle of transcript assembly in the future. This study illustrates the power of comparative transcriptomics and presents a new correlation-based approach useful to analyze this type of data.

## Author contributions

IF, KA, EP, EAnd and EAlex planned the study. KA performed the lab experiment with assistance of EAlex. Bioinformatics analysis was done by IF and EP. All authors interpreted the data, wrote and approved the final manuscript.

### Conflict of interest statement

The authors declare that the research was conducted in the absence of any commercial or financial relationships that could be construed as a potential conflict of interest.

## Supplementary material

Figure S1 **QC plots for the species and clones**. *S. nigrum* **(A)**, *S. dulcamara* **(B)**, *S. physalifolium* **(C)**, Sarpo Mira **(D)**, SW93-1015 **(E)**, and Desiree **(F)**. For each basepair position a Box-and-Whisker plot is drawn. The y-axis on the graph shows the quality scores. The higher the score the better the base call. The background of the graph divides the y-axis into calls of very good quality (green), calls of reasonable quality (orange), and calls of poor quality (red).

Figure S2 **Clades of GO enriched terms for Biological Process (Siddappa et al., 2014) in of the expanded and depleted OrthoMCL clusters determined qualitatively (resistant vs. susceptible)**. Broad, unspecific GO terms have been removed.

Figure S3 **Venn diagram showing the overlap of the OrthoMCL clusters containing identified R-genes in the studied ***Solanum*** species and potato clones**.

Figure S4 **Overlap of ***P. infestans*** transcripts detected in the wild ***Solanum*** species and potato clones**.

Table S1 **Transcript numbers for the ***Solanum*** species and potato clones for each OrthoMCL cluster including ***p***-values (Spearman's rho test) for the degree of quantitative and qualitative positive or negative association**.

Table S2 **OrthoMCL clusters with transcript PGSC identifiers**.

Table S3 **NB-LRR-specific amino acid motifs identified with psp-gen MEME**. Motif number, motif sequence, domain group (TNL or CNL) and whether the motif is similar to a known motif are indicated.

Table S4 **Transcripts of Desiree, ***S. nigrum*** and ***S. physalifolium***, ***S. dulcamara***, Sarpo Mira, and SW93-1015 identified by MEME**. The classification (TNL or CNL) is provided as well as NBS-LRR motif validation by pfam. For the potential R-gene transcripts the OrthoMCL cluster ID is given as well as number of transcripts in the clusters and *p*-values (Spearman's rho test) for the degree of quantitative and qualitative association.

Table S5 **Potential ***R***-gene homolog transcripts in Desiree, ***S. nigrum*** and ***S. physalifolium***, ***S. dulcamara***, Sarpo Mira, and SW93-1015 identified by BLASTP searches against the Plant Resistance Genes database**. The classification is provided as well as NBS-LRR motif validation by pfam. For the potential R-gene transcripts the OrthoMCL cluster ID is given as well as number of transcripts in the clusters and *p*-values (Spearman's rho test) for the degree of quantitative and qualitative association.

Table S6 **Putative ***R1***, ***R2***, ***R3***, and ***blb1*** transcripts in Desiree, ***S. nigrum*** and ***S. physalifolium***, ***S. dulcamara***, Sarpo Mira, and SW93-1015 identified by BLASTP and BLASTN searches with ***R1***, ***R2***, ***R3***, and ***blb1*** representatives**. For the potential R-gene transcripts the OrthoMCL cluster ID is given as well as number of transcripts in the clusters and *p*-values (Spearman's rho test) for the degree of quantitative and qualitative association.

Table S7 *****S***-gene homologs among the transcripts in the ***Solanum*** wild species and clones identified by BLASTP searches using ***A. thaliana*** mitogen-activated protein kinase 4 (MAPK 4), ***A. thaliana*** homoserine kinase 1 and 6 (dmr1, dmr6) and Barley mlo and its Capsicum and ***S. tuberosum*** homologs**.

Table S8 **The number of transcripts of CRN, RxLR effector and elicitin in Desiree, ***S. nigrum*** and ***S. physalifolium***, ***S. dulcamara***, Sarpo Mira and SW93-1015 together with the accession ID and annotation**.

Table S9 **(A)** GO terms enriched in unique OrthoMCL clusters in Desiree, *S. nigrum* and *S. physalifolium*, *S. dulcamara*, Sarpo Mira and SW93-1015. **(B)** GO terms enriched in unique OrthoMCL clusters in potato clones vs. *Solanum* wild species. The hypergeometic tests *p*-value are provided.
