# Supplementary material for: A novel workflow correlating RNA-seq data to Phythophthora infestans resistance levels in wild Solanum species and potato clones
Source: Front Plant Sci. 2015 Sep 17;6:718. doi: 10.3389/fpls.2015.00718 (PMC4585127; doi:10.3389/fpls.2015.00718)
Supplement: Figure S1 — QC plots for the species and clones. S. nigrum (A), S. dulcamara (B), S. physalifolium (C), Sarpo Mira (D), SW93-1015 (E), and Desiree (F). For each basepair position a Box-and-Whisker plot is drawn. The y-axis on the graph shows the quality scores. The higher the score the better the base call. The background of the graph divides the y-axis into calls of very good quality (green), calls of reasonable quality (orange), and calls of poor quality (red). [file DataSheet1.DOCX]

**Supplementary material**

Title: **A novel workflow correlating RNA-seq data to resistance levels of wild *Solanum* species and potato clones to *Phytophthora infestans***

Authors: Itziar Frades^1^*, Kibrom B. Abreha^1^*, Estelle Proux-Wéra^,1,2^, Åsa Lankinen^1^, Erik Andreasson^1^, Erik Alexandersson^1^

*joint first authors

^1^Deptartment of Plant Protection Biology, Swedish University of Agricultural Sciences in Alnarp, Sweden

^2^Current affiliation: SciLifeLab, Department of Biochemistry and Biophysics, Stockholm University

Corresponding author:

Erik Alexandersson

email: [erik.alexandersson@slu.se](mailto:erik.alexandersson@slu.se), tel: +46-40-415338


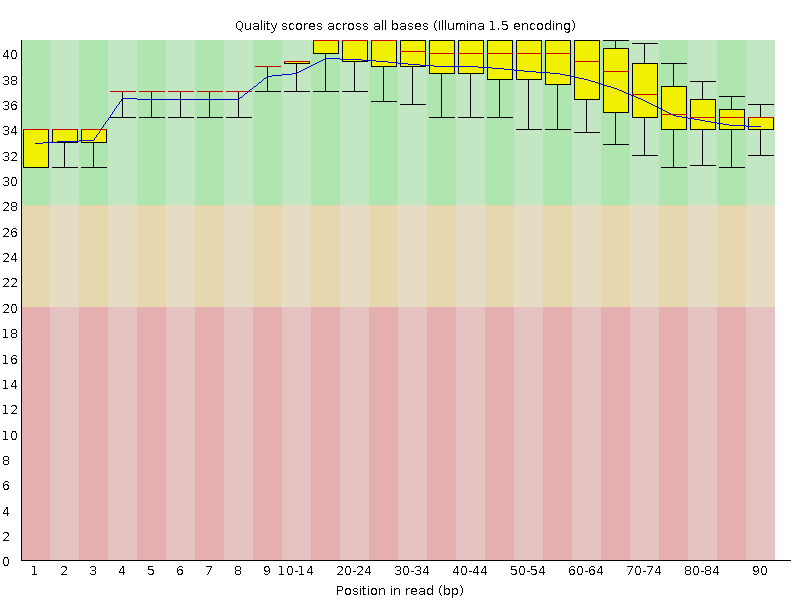

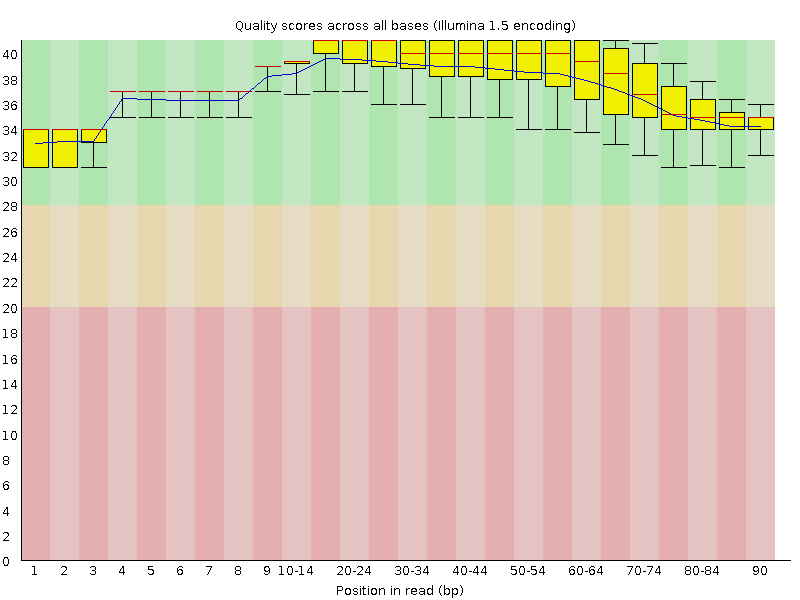


**C**

**B**

**A**


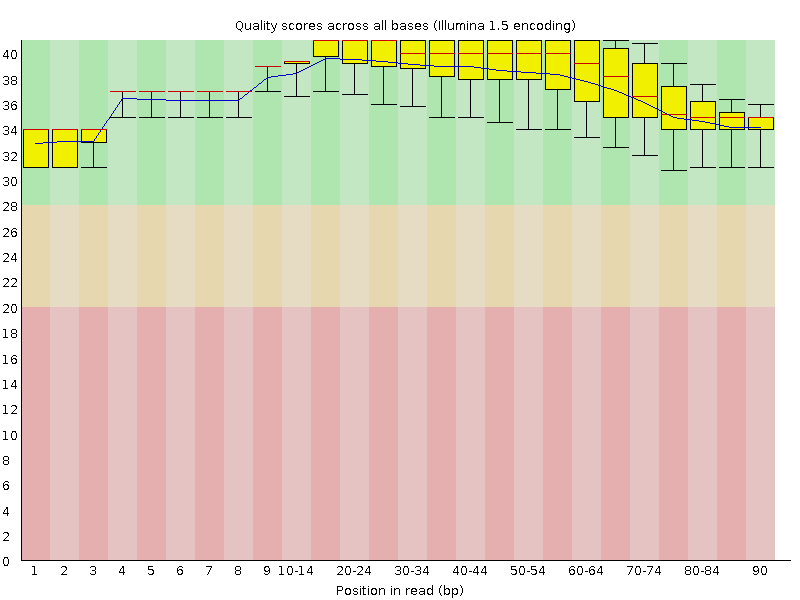

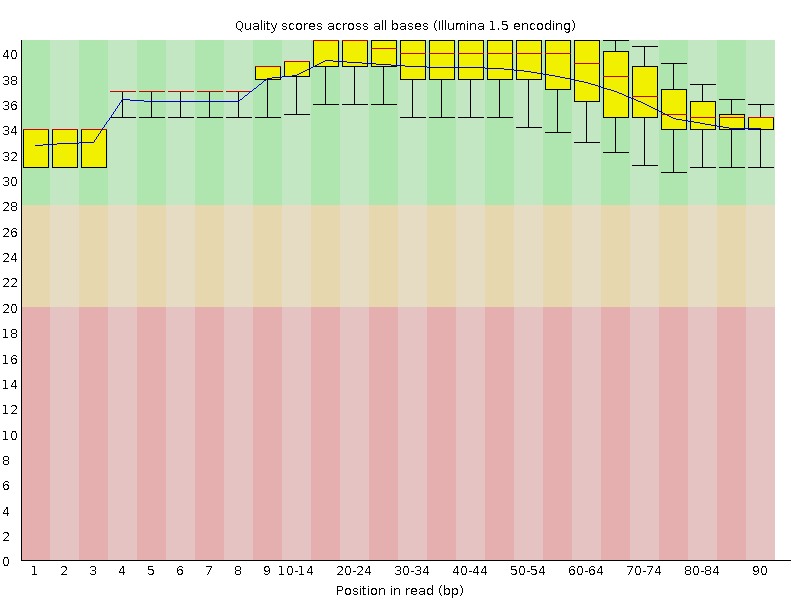


**D**


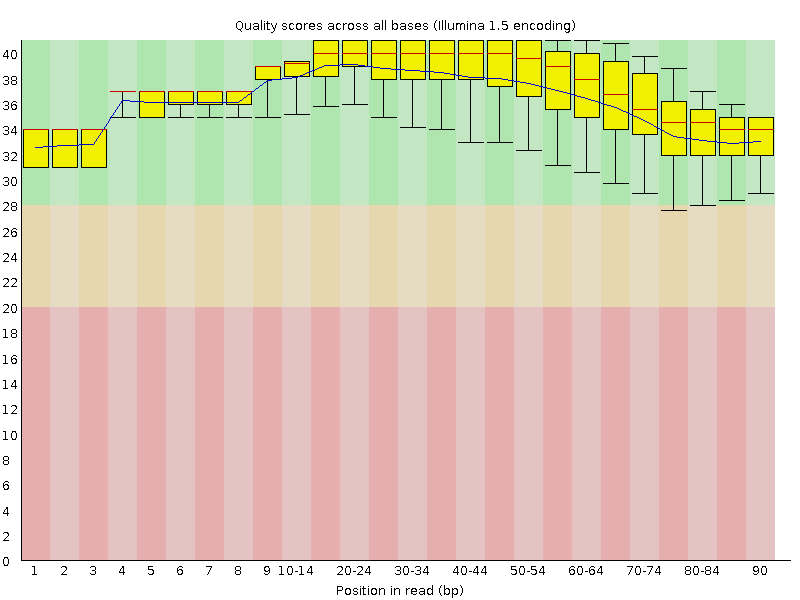

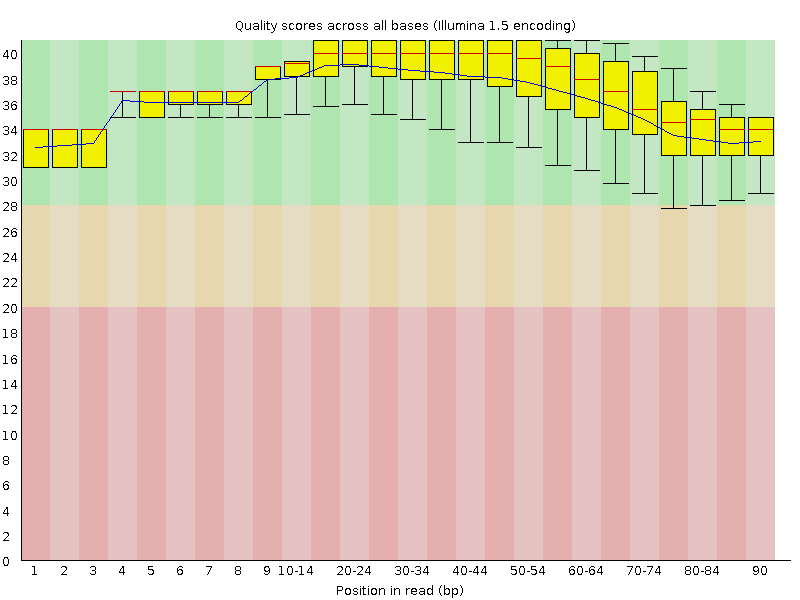


**F**

**E**

Figure S1: QC plots for S. nigrum (A), S. dulcamara (B), S. physalifolium (C), Sarpo Mira (D), SW93-1015 (E), and Desiree (F). For each position a BoxWhisker type plot is drawn. The y-axis on the graph shows the quality scores. The higher the score the better the base call. The background of the graph divides the y axis into very good quality calls (green), calls of reasonable quality (orange), and calls of poor quality (red).


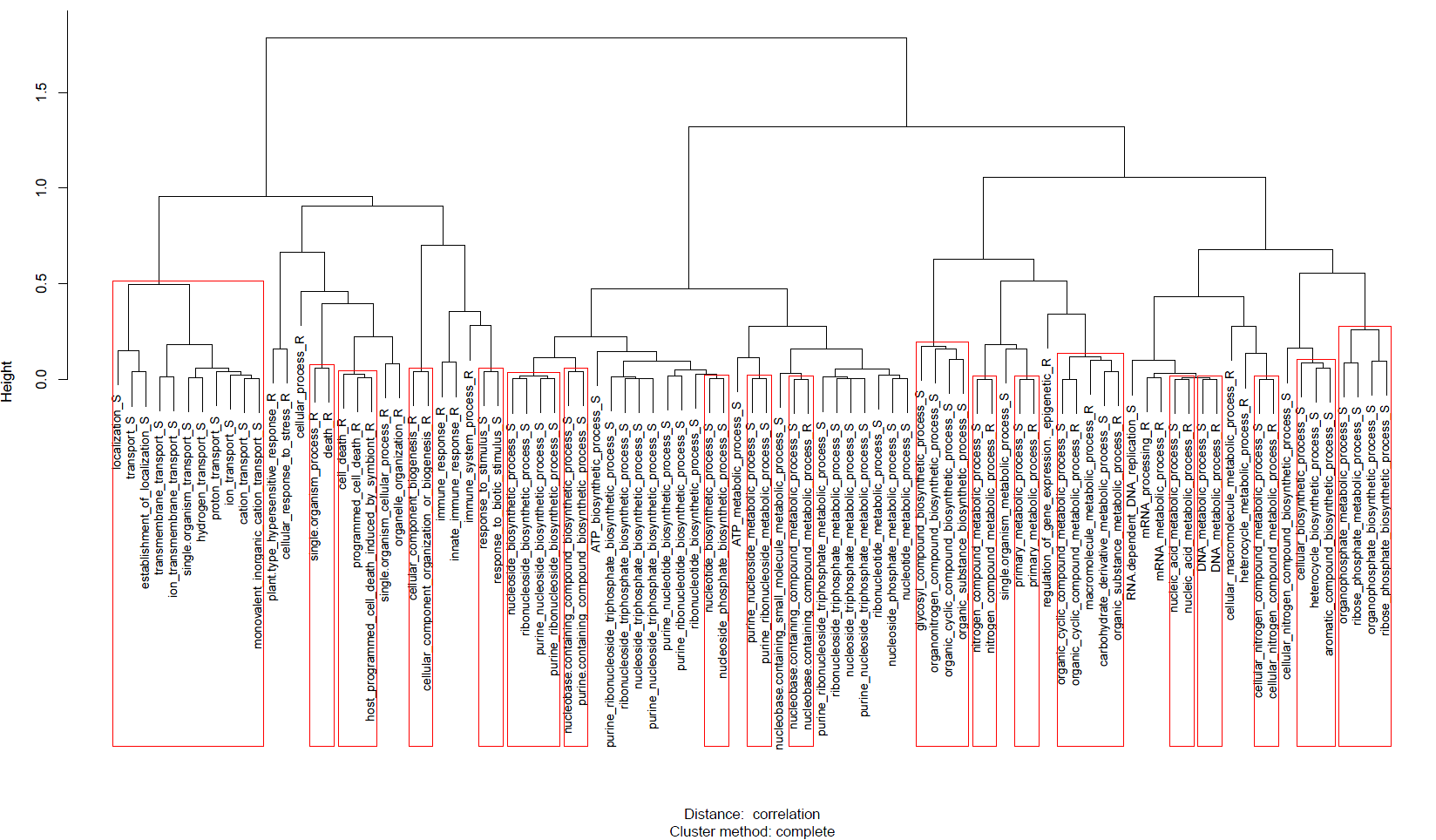


Figure S2: Clades of GO enriched terms for Biological Process (BP) in of the expanded and depleted OrthoMCL clusters determined qualitatively (resistant vs. susceptible). Broad, unspecific GO terms have been removed.


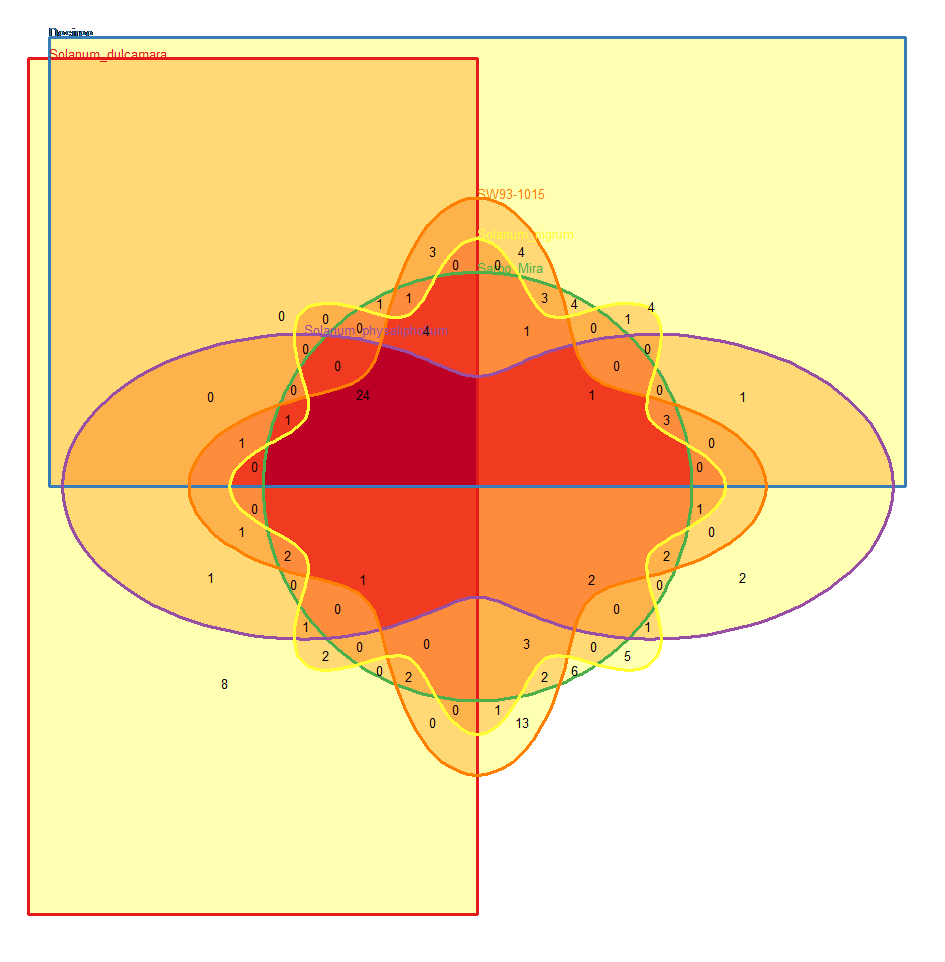


Figure S3: Venn diagram showing the overlap in the studied *Solanum* lineages of the OrthoMCL clusters where the R-genes lie.


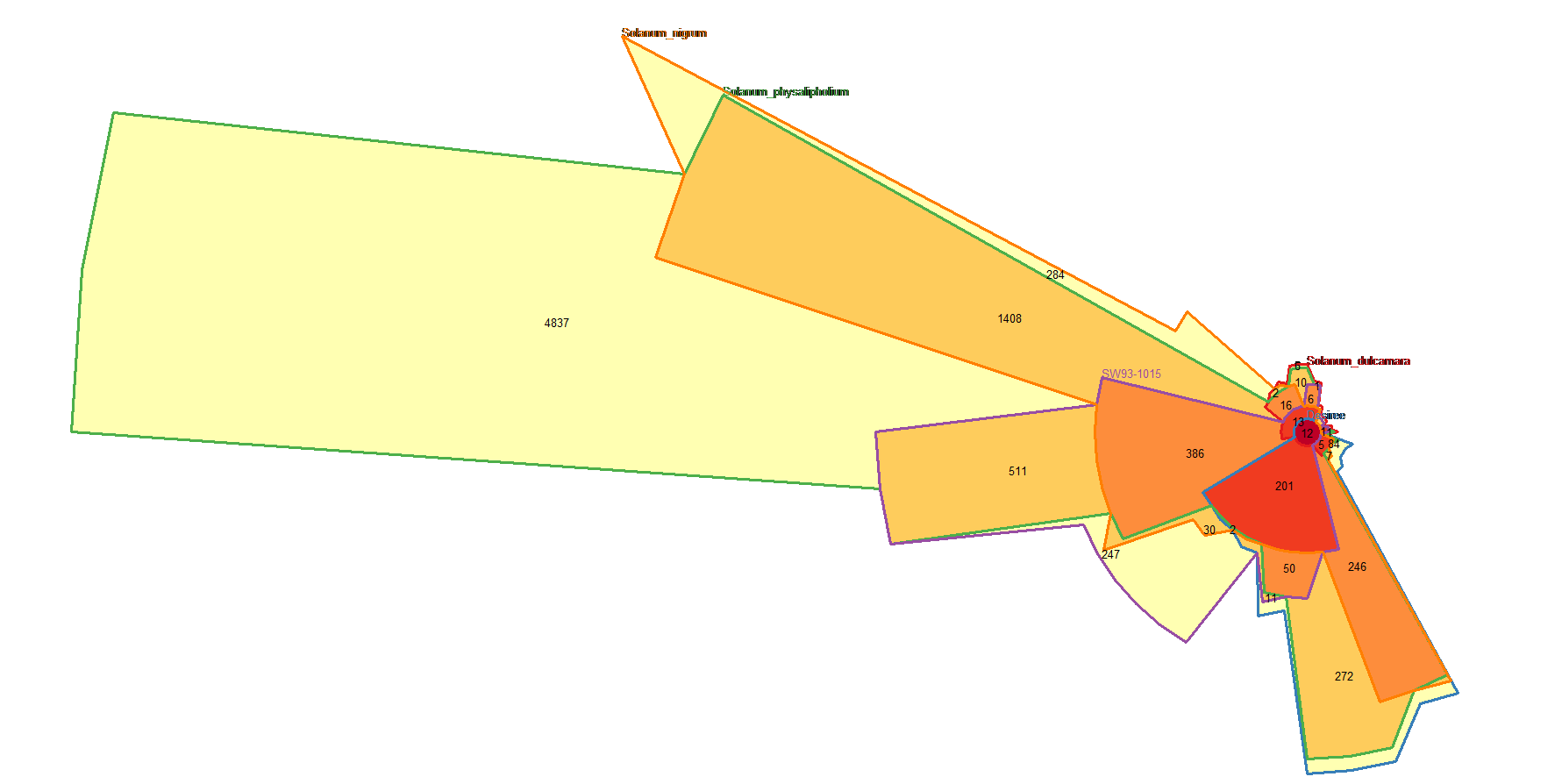


Figure S4: Overlap of *P. infestans* transcripts detected for respective wild *Solanum* species and potato clone.
